# Genome-Wide Identification of Wild Soybean Mitochondrial Calcium Uniporter Family Genes and Their Responses to Cold and Carbonate Alkaline Stresses

**DOI:** 10.3389/fpls.2022.867503

**Published:** 2022-05-03

**Authors:** Jianwei Li, Mingzhe Sun, Yu Liu, Xiaoli Sun, Kuide Yin

**Affiliations:** ^1^Crop Stress Molecular Biology Laboratory, College of Agriculture, Heilongjiang Bayi Agricultural University, Daqing, China; ^2^Key Laboratory of Soybean Biology of Chinese Education Ministry, Northeast Agricultural University, Harbin, China

**Keywords:** wild soybean, mitochondrial calcium uniporter, cold stress, carbonate alkali stress, expression analysis

## Abstract

The mitochondrial calcium uniporter (MCU), as an important component of the Ca^2+^ channel uniporter complex, plays a regulatory role in intracellular Ca^2+^ signal transduction. However, only a few studies to date have investigated plant MCU genes. In this study, we identified the MCU family genes in wild soybean and investigated their expression under cold and carbonate alkaline stresses. Eleven *Glycine soja* MCU genes (*GsMCUs*) were identified and clustered into two subgroups (subgroups I and II), and subgroup II could be further divided into two branches (MCU5 and MCU6). A total of 21 pairs of *GsMCUs* were characterized as duplicated genes, and displayed a similar exon-intron architecture. All GsMCU proteins contained one conserved MCU domain, within which two transmembrane domains were found. An analysis of the conserved motifs further supported that the GsMCUs showed high conservation in protein sequence and structure. Moreover, we found that all *GsMCUs* were expressed ubiquitously in different tissues and organs, and *GsMCU*s from the same subgroup displayed varied tissue expression profiles. In addition, based on RNA-seq and qRT-PCR assays, six and nine *GsMCUs* were differentially expressed under cold and carbonate alkaline stress, respectively. Promoter analysis also uncovered the existence of two canonical cold-related *cis*-acting elements, LTR and DRE/CRT, as well as stress-related phytohormone-responsive elements. Our results provide valuable information about the MCU family in soybean responses to cold and carbonate alkaline stress, which will be helpful in further characterizing their biological roles in response to abiotic stress.

## Introduction

In the 1970s, calcium ions were proposed to be important messenger ions in cells. Over the years, a large number of studies have demonstrated that many functions of cells are inextricably linked to Ca^2+^ (Clapham, 2007). Mitochondria absorb Ca^2+^ mainly through mitochondrial calcium uniporters (MCU) located on the inner membrane (Deluca and Engstrom, 1961; Vasington and Murphy, 1962). A typical MCU protein harbors two transmembrane domains within a conserved MCU domain (PF04678), with the N- and C-terminus facing the mitochondrial matrix. The N-terminal domain is essential for the Ca^2+^ uptake ability of MCU proteins, while the formation of a functional channel requires the pentamerization of transmembrane regions. By interacting with MICU1 (mitochondrial calcium uptake 1), MCUs can transport Ca^2+^ along electrochemical gradients (Perocchi et al., 2010), and play key roles in mitochondrial Ca^2+^ homeostasis, regulation and signaling (Baughman et al., 2011; De Stefani et al., 2011; Drago et al., 2011). In plants, MCUs are important components for sensing environmental stress because they modulate stromal Ca^2+^ in chloroplasts (Teardo et al., 2019). In recent decades, an increasing number of MCUs have been functionally characterized in mammals (Mallilankaraman et al., 2012; Bisbach et al., 2020; Liu et al., 2020), but little has been reported regarding plant MCUs.

Taking advantage of whole genome sequencing and public expression data, researchers could genome-widely identify MCU family genes, and systemically investigate their expression characteristics, which will provide hints to deeply study the function of MCUs in diverse physiological and biological processes. However, compared with other gene families, fewer studies have been reported on MCU family. To date, six, five, seven, and four MCUs have been identified in *Arabidopsis thaliana* (Stael et al., 2012), *Oryza sativa* (Gu, 2020), *Pyrus seratina* (Wang et al., 2018), and *Sorghum bicolor* (Gu, 2020), respectively. Among the six MCU family genes in Arabidopsis, *AtMCU1* and *AtMCU2* were found to regulate pollen tube germination and growth (Selles et al., 2018), while *AtMCU6*, a chloroplast-localized mitochondrial calcium uniporter, negatively modulated resistance to long-term water deficits (Teardo et al., 2019). In pears, MCUs have been suggested to play an important role in fruit ripening and senescence (Wang et al., 2018). However, there have been no reports on soybean MCU proteins.

Compared with cultivated soybean (*Glycine max* L.), wild soybean (*Glycine soja*) exhibits very high adaptability to harsh environments and extensive genetic diversity (Kofsky et al., 2018). It will be of great help to identify the soybean MCU genes, and screen the stress responsive MCUs in wild soybean. Therefore, in this study, we systematically characterized the MCU family genes in wild soybean and investigated their expression profiles in different tissues and under abiotic stress. Our findings suggest the potential regulatory roles of wild soybean MCU genes in response to cold and carbonate saline-alkaline stresses, which will facilitate the molecular breeding of soybean cultivars with higher tolerance to cold and carbonate saline-alkaline stresses.

## Materials and Methods

### Identification of the Mitochondrial Calcium Uniporter Family Genes in the Wild Soybean Genome

To identify the candidate MCU genes in the wild soybean genome, we obtained the MCU proteins sequences which have been identified in *Arabidopsis thaliana* (Stael et al., 2012), *Pyrus seratina* (Wang et al., 2018), *Oryza sativa* and *Sorghum bicolor* (Gu, 2020) from Phytozome database.^1^ These protein sequences were used for generating a hidden Markov Model,^2^ which was then used to search against the wild soybean (*Glycine soja v1.1*) and cultivated soybean (*Glycine max Wm82.a2.v1*) proteomes. The amino acid sequences of the candidates were manually checked to determine whether they harbored the MCU domain (PF04678) using the NCBI Conserved Domain Database^3^ and Pfam^4^ program.

Detailed information on the GsMCUs, including their locus ID and genomic and protein sequences, was obtained from the Phytozome database. Isoelectric point values and molecular weights were estimated using ExPASy.^5^ The transmembrane (TM) region was predicted by using the TMHMM.^6^

### Chromosomal Distribution and Gene Duplication Analysis

The chromosomal locations of the GsMCUs were obtained from the Phytozome database. Duplicated segmental blocks of soybean genome were downloaded from the Plant Genome Duplication Database (PGDD). The criteria for identifying duplicated gene pairs were: (a) the length of the shorter aligned sequence covered > 70% of the longer sequence; and (b) the similarity of the two aligned sequences were > 70%. Orthologous soybean genes were downloaded from Ortho Venn2.^7^ The chromosomal locations and syntenic information were visualized using TBtools software. Ka (non-synonymous rate), Ks (synonymous rate), and Ka/Ks ratios were calculated using TBtools according to their coding sequences. The timing of the genome-wide duplication event was calculated using the formula: Time = Ks/2r (*r* = 6.98 × 10^–9^).

### Phylogenetic Relationship, Exon-Intron Organization and Protein Structure Analysis

For phylogenetic analysis, multiple alignments were performed with full-length amino acid sequences of MCU proteins in rice, Arabidopsis, and soybean using ClustalW. A neighbor-joining phylogenetic tree was constructed using MEGA5.1 software with the following parameters: Poison correction, pairwise deletion, uniform rates, and 1,000 bootstrap replicates.

The exon/intron structure of the GsMCU genes was analyzed using the Gene Structure Display Server (GSDS).^8^ Functional domain information was derived from the NCBI Batch-CDD database.^9^ The conserved motifs were predicted using MEME^10^ with the following parameters: number of motifs = 15 and zero or one occurrence per sequence. The functional domains and conserved motifs of the GsMCUs were illustrated using TBtools.

### Protein Interaction Network Analysis

The protein-interacting network of the GsMCUs was generated using the STRING database.^11^

### *Cis*-Acting Elements Analysis

The 2,000 bp promoter sequences upstream of the transcript start sites of the *GsMCU* genes were obtained from the Phytozome database (see text footnote 1). The *cis*-acting elements were analyzed using New PLACE.^12^

### Accession Numbers of Public Expression Data

The accession numbers of the public expression data are as follows: NaHCO_3_ stress (GSE17883), and cold stress (GSE117686) (Yamasaki and Randall, 2016; Robison et al., 2019).

### Plant Growth Conditions and Stress Treatments

*Glycine soja* (G07256) seeds were treated with 98% sulfuric acid for 10–15 min, and washed five times with distilled water, and then kept in complete darkness with humidity for 1 day to promote germination. The seedlings were transferred and grown in 1/2 Hoagland’s nutrient solution at 26–28°C under 16-h light/8-h dark conditions.

To explore the gene expression patterns in different tissues, freshly germinated seeds, roots, stems, and leaves of 21-day-old seedlings and the flowers were harvested. For the cold stress, 2-week-old seedlings were exposed to 4°C. For the carbonate saline-alkaline treatment, 21-day-old seedlings were transferred to a Hoagland’s nutrient solution containing 50 mM NaHCO_3_ (pH 8.5). Equal amounts of seedling roots were harvested at the indicated time points, and the samples were snap-frozen in liquid nitrogen and stored at −80°C.

### RT-PCR Assays

The total RNA was isolated using the TriPure Isolation Reagent (Roche, United States), and cDNAs were synthesized using HiScript ^®^ III RT SuperMix for qPCR (Vazyme, Nanjing, China) according to the manufacturer’s instructions. Quantitative real-time PCR assays were performed using *TransStart* ^®^ Top Green qPCR SuperMix according to the manufacturer’s protocol. The F_box gene in wild soybeans (GlysoPI483463.12G046600) was used as an internal control (Le et al., 2012; Bansal et al., 2015). All experiments were performed using three independent biological replicates for statistical analyses. The primers used are listed in Supplementary Table 3.

## Results

### Identification of Mitochondrial Calcium Uniporter Family Members in Wild Soybean

To investigate the precise information and potential function of *Glycine soja* MCU (*GsMCU*) genes, we carried out systematic genome-wide identification of putative MCU genes in the wild soybean genome. To do this, we generated a hidden Markov model based on protein sequences of the reported Arabidopsis (Stael et al., 2012), *Pyrus seratina* (Wang et al., 2018), and *Sorghum bicolor* (Gu, 2020) MCU proteins, respectively, and then searched for MCU candidates against the soybean proteome. After manually checking protein sequences for to ensure the existence of the MCU domain, a total of 11 genes were identified as GsMCU family members and named according to Arabidopsis orthologs.

Detailed information about wild soybean (*Glycine soja* v1.1) GsMCUs was obtained from the Phytozome database and is listed in Table 1. Notably, GsMCU5.2 had only 264 amino acids (aa), and did not possess the N-terminal extension, compared to other members. The other GsMCU members shared similar protein sequence lengths (from 317 to 357 aa). Correspondingly, the molecular weight of the GsMCU proteins (except GsMCU5.2) varied from 36.2 to 40.6 kDa, and the predicted isoelectric points ranged between 8.65 and 9.51. Furthermore, all GsMCU proteins had two predicted transmembrane regions, with both the N-and C-terminus facing the matrix. These results imply that the GsMCU family possesses a conserved protein structure.

**TABLE 1:** Detailed information of the MCU family genes in wild soybean.

			Genomic sequence length (bp)	Protein			TM
No.	Gene name	TIGR locus ID		Amino acids (aa)	Isoelectric point	Molecular weight (KD)	
1	*GsMCU1.1*	GlysoPI483463.14G165600	4,011	339	8.8	38.13	2
2	*GsMCU1.2*	GlysoPI483463.02G198500	2,646	346	8.9	38.5	2
3	*GsMCU1.3*	GlysoPI483463.11G188100	3,224	328	9.02	37.09	2
4	*GsMCU1.4*	GlysoPI483463.18G044900	2,681	338	8.65	37.87	2
5	*GsMCU5.1*	GlysoPI483463.14G067700	2,677	348	9.35	39.87	2
6	*GsMCU5.2*	GlysoPI483463.17G212600	3,031	264	9.01	30.79	2
7	*GsMCU5.3*	GlysoPI483463.17G212700	2,645	357	9.24	40.6	2
8	*GsMCU6.1*	GlysoPI483463.01G141000	2,217	325	9.11	36.66	2
9	*GsMCU6.2*	GlysoPI483463.11G058600	2,876	325	9.24	36.81	2
10	*GsMCU6.3*	GlysoPI483463.02G052500	4,547	317	9.51	36.2	2
11	*GsMCU6.4*	GlysoPI483463.16G112200	4,982	328	9.17	37.59	2

### Phylogenetic Relationship and Structure Analysis of *Glycine soja* MCU Genes

**FIGURE 1:**
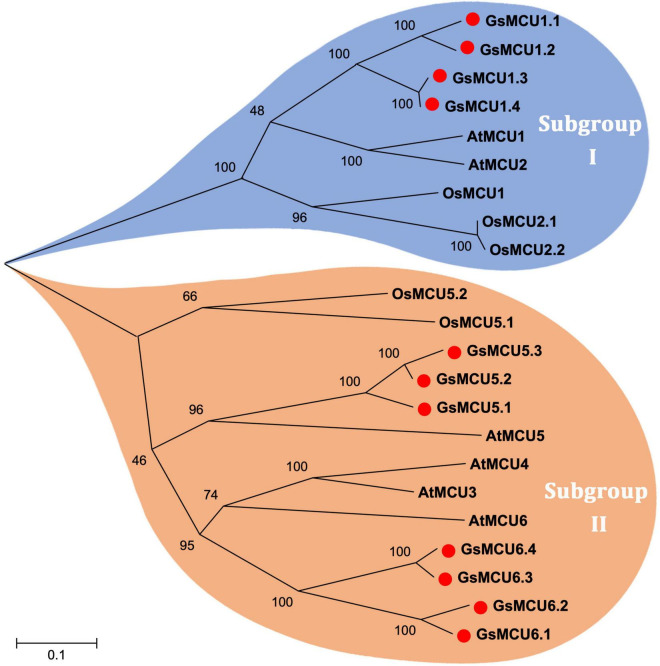
Phylogenetic relationship of MCU proteins from Arabidopsis, rice and soybean. The phylogenetic tree was constructed using MEGA 5.10 software using the neighbor-joining method, and the bootstrap values were set at 1,000.

To investigate the phylogenetic relationships among the GsMCUs, we generated a neighbor-joining tree using the full-length amino acid sequences of wild soybean, Arabidopsis (Stael et al., 2012) and rice (Gu, 2020) MCU family proteins (Figure 1). As shown in Figure 1, MCU proteins were clustered into two subgroups: subgroup I and subgroup II. Subgroup I contained nine members, including four GsMCUs, two AtMCUs, and three OsMCUs, whereas subgroup II included seven GsMCUs, four AtMCUs, and two OsMCUs (Supplementary Table 1). In each subgroup, the OsMCUs were first separated from the other members and clustered into one branch. This finding implies that the appearance of these two subgroups occurred before the division of monocots and dicots during evolution. Moreover, the soybean possessed more MCU genes than Arabidopsis and rice, and GsMCUs appeared in pairs in the phylogenetic tree, which might be a consequence of whole genome duplication of soybean during evolution. This result also indicates possible gene duplication within the GsMCU family, as well as the conservation of GsMCUs in gene architecture and protein structure.

### Gene Duplication and Structure Analysis of *Glycine soja* MCUs

**FIGURE 2:**
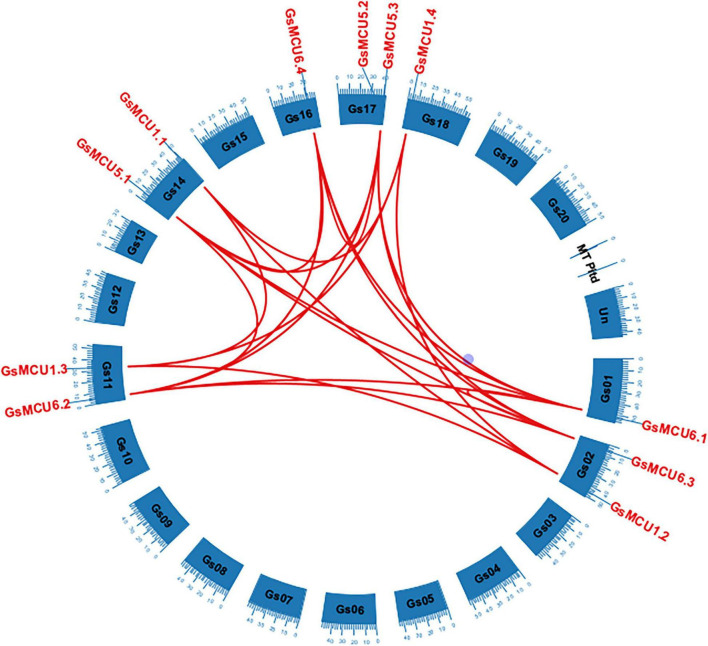
Distribution and duplication of the MCU genes in the wild soybean genome. Duplicated genes among the GsMCUs were downloaded from the PGDD database and are indicated by red lines.

To confirm the existence of gene duplication within the GsMCU family, we performed chromosomal location and collinearity analyses. As shown in Figure 2, 11 *GsMCUs* were randomly distributed on seven chromosomes. Chromosomes 2, 11, 14, and 17 each possessed two *GsMCUs*, whereas chromosomes 1, 16, and 18 each contained one. To obtain the collinearity of GsMCUs, we retrieved duplicated gene pairs of the MCU family from the Plant Genome Duplication Database and identified 21 pairs among 11 GsMCU genes (Figure 2). Furthermore, we found that the Ka/Ks values of the duplicated pairs were less than 1, indicating purification of GsMCU genes during evolution (Supplementary Table 2). In addition, the duplication of the GsMCUs occurred 10.5246–322.3279 Mya (million years ago).

**FIGURE 3:**
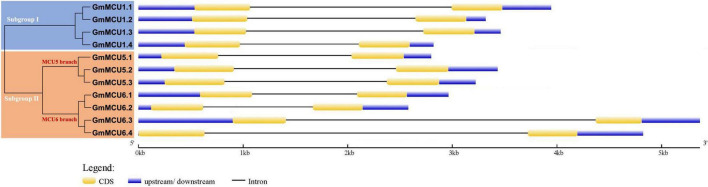
Gene structure analysis of GsMCU family. The position of exons, introns, and untranslated regions (UTRs) are indicated by yellow, black, and blue boxes, respectively. The scale bar at the bottom was used to estimate the sizes of the exons, introns, and untranslated regions.

In generally, duplicated gene pairs are highly conserved in terms of exon-intron organization. As shown in Figure 3, all *GsMCUs* contained only two exons of similar length, suggesting a highly conserved gene architecture. Moreover, synteny analysis of MCU genes from different dicot species identified a number of duplicated MCU gene pairs among soybean, alfalfa, and Arabidopsis (Supplementary Figure 1). These findings further support the idea that whole-genome duplication was the main contributor to expansion of the MCU family.

### Conserved Domains of *Glycine soja* MCU Proteins

**FIGURE 4:**
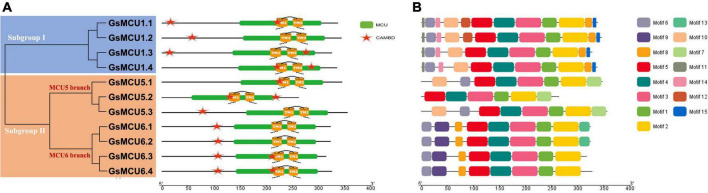
Functional domains and conserved motifs of GsMCU proteins. Functional domains **(A)** and conserved motifs **(B)** are indicated by green boxes and different colored boxes represent different motifs. The five-pointed stars represent the calmodulin-binding sites in the GsMCU proteins. Protein domains and conserved motifs were predicted using a CDD search and MEME.

**FIGURE 5:**
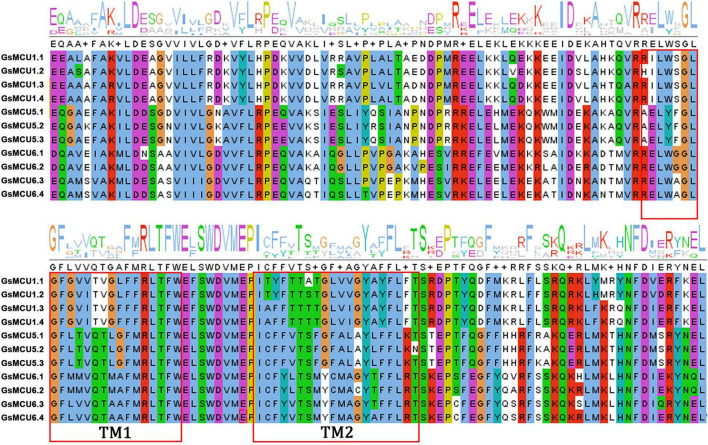
Multiple sequence alignment of the MCU domain within GsMCUs proteins. Two transmembrane regions (TM1 and TM2) are marked by red boxes.

To verify the conservation of the GsMCU family, we further analyzed the functional domains and conserved motifs. All GsMCUs harbored one conserved MCU domain (PF04678) (Figure 4A). MEME analysis of the conserved motifs also suggested that the GsMCU proteins possessed high similarity in motif organization (Figure 4B). Among the 15 motifs, motifs 1 to 4, representing the MCU domain, were present in all GsMCUs. Multiple alignments illustrated that the MCU domain contained two transmembrane regions (TMs) and displayed a high sequence similarity (Figure 5). These results further highlight the high conservation of GsMCUs in terms of protein structure.

Meanwhile, GsMCUs from different subgroups also showed sequence diversity at a certain degree (Figure 4B). For example, among the 15 motifs, motifs 11, 14, and 15 were specific to subgroup I GsMCUs. Motif 9 was only observed in the MCU6 branch of subgroup II, whereas motif 7 was observed in the MCU5 branch. Interestingly, motif 8 was located at the C-terminus of subgroup I, but at the N-terminus of the subgroup II MCU6 branch. In addition, one or two calmodulin-binding domains (CAMBD) were found in all GsMCU proteins. However, the location of the CAMBD varied among the different GsMCU proteins (Figure 4A). These differences imply possible different regulatory mechanisms of biochemical activity or functional specialization.

### Tissues Expression Profiles of *Glycine soja* MCU Genes

**FIGURE 6:**
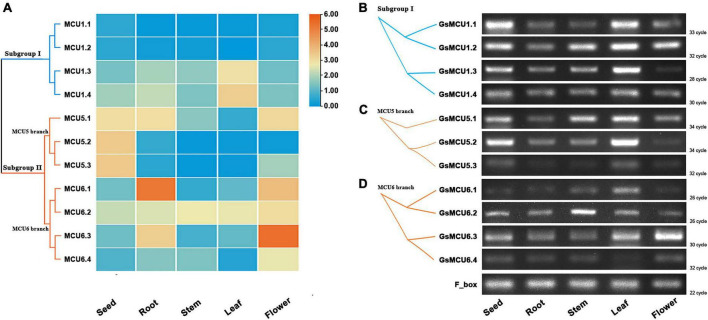
Expression of GsMCU genes in different tissues. **(A)** Heat map showing the expression levels of soybean MCUs in different tissues. The color scale represents the expression values: red indicates high levels and blue represents low levels. **(B–D)** RT-PCR assays for *GsMCUs* expression in different tissues. F_box (GlysoPI483463.12G046600) was used as an internal reference.

Several studies have reported the involvement of plant MCU genes in growth and development (Wang et al., 2018). To explore the potential function of MCU genes in soybean growth, we retrieved the expression profiles of GsMCUs in different tissues from Phytozome and verified these profiles via semiquantitative RT-PCR analysis (Figure 6). According to our RT-PCR results, the GsMCU genes were expressed in all detected tissues and organs (Figures 6B–D). For the four GsMCUs in subgroup I, two (*GsMCU1.1* and *GsMCU1.2*) were expressed at a very low level, and the other two (*GsMCU1.3* and *GsMCU1.4*) displayed a relatively high expression level, with the highest expression in leaves (Figure 6A). The RT-PCR results confirmed this pattern (Figure 6B). For members of the MCU5 branch in subgroup II, *GsMCU5.1* was highly expressed in roots, flowers, and seeds, while *GsMCU5.2* and *GsMCU5.3* displayed high expression in seeds (Figure 6A). The RT-PCR analysis revealed a relatively high expression of the MCU5 branch genes in seeds and leaves (Figure 6C). This finding indicates a possible role for the MCU5 branch in seed development. In the MCU6 branch, GsMCU6.1 displayed the highest expression in roots, while the other three genes showed the highest expression levels in flowers (Figure 6A). As shown in Figure 6D, *GsMCU6.1* showed a peak expression in leaves, *GsMCU6.2* displayed a ubiquitous expression, with the highest level in the stem, while GsMCU6.3 and *GsMCU6.4* had the highest transcript levels in flowers. In conclusion, these results suggest that *GsMCU*s from the same subgroup display variable tissue expression profiles, indicating their potentially divergent roles in soybean development.

### Expression Patterns of *Glycine soja* MCU Under Cold and Carbonate Alkaline Treatments

To date, few studies have reported the function of plant MCUs in abiotic stress response. Considering the severe effects of low temperature and saline-sodic stress on soybean yields, we further analyzed the expression patterns of GsMCUs under cold and carbonate alkaline treatments to explore their potential roles in these stress responses.

**FIGURE 7:**
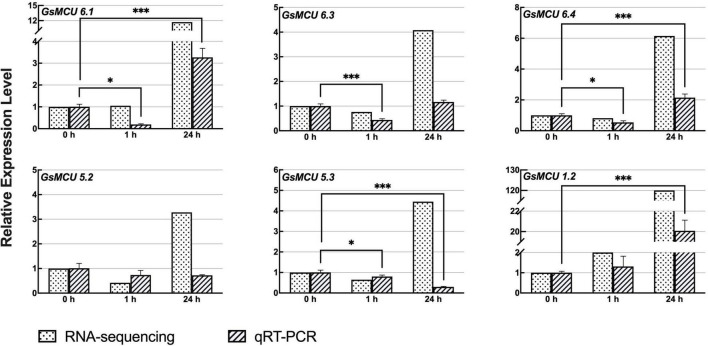
Expression analyses of wild soybean MCU family genes under cold stress. To induce cold stress, 2-week-old wild soybean seedlings were treated at 4°C for 0, 1, and 24 h. GlysoPI483463.12G046600 was used as an internal reference. Mean values (±SE) of the three fully independent biological repeats and three technical repeats are shown. Asterisks indicate significant differences (*p < 0.05, ^***^p < 0.001 using Student’s *t*-test).

Previous RNA-seq data (Yamasaki and Randall, 2016; Robison et al., 2019) showed that six members of the soybean MCU family genes responded to cold treatment (Figure 7). To confirm the differential expression of the *GsMCUs* after cold treatment, we performed quantitative real-time PCR assays and found that the expression of three MCU6 branch genes (*GsMCU6.1*, −*6.3*, −*6.4* in subgroup II) decreased at 1 h and then increased at 24 h after cold stress. However, the expression of *GsMCU5.2* and *GsMCU5.3* from the MCU5 branch in subgroup II was repressed by cold stress. Furthermore, in subgroup I, *GsMCU1.2* expression was greatly increased at 24 h of cold treatment. These expression data imply that these *GsMCUs* may play different roles in cold stress response.

**FIGURE 8:**
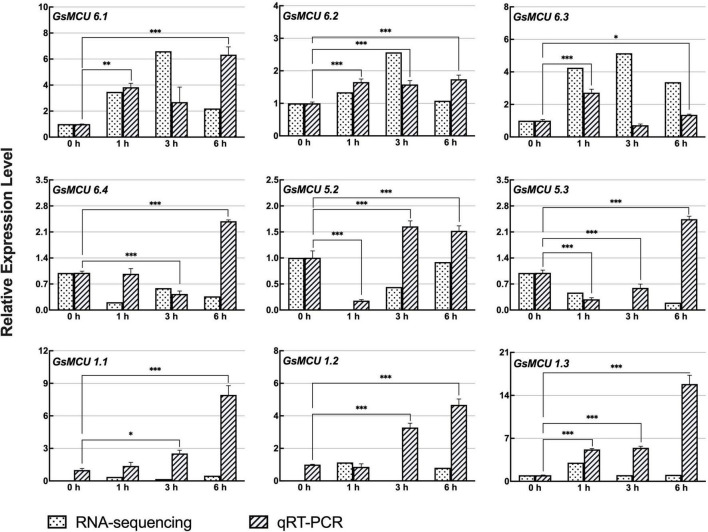
Expression analyses of wild soybean MCU family genes under NaHCO_3_ stress. For NaHCO_3_ stress, the 21-day-old wild soybean seedlings were treated with 50 mM NaHCO_3_ for 0, 1, 3, and 6 h. GlysoPI483463.12G046600 was used as an internal reference. The mean values (±SE) from three fully independent biological repeats and two technical repeats are shown. Asterisks indicate significant differences (*p < 0.05, ^**^p < 0.01, ^***^p < 0.001 by Student’s *t*-test).

According to our previous RNA-seq data, nine *GsMCUs* showed increased expression after carbonate alkaline treatment (50 mM NaHCO_3_, pH 8.5). As shown in Figure 8, qRT-PCR results showed that the expression of these nine *GsMCUs* was altered by carbonate alkaline treatment. In details, the expression of four genes (*GsMCU6.1*, −*6.2*, −*1.1*, and −*1.3*) was significantly up-regulated at all detected time points after NaHCO_3_ treatment. *GsMCU5.2* and *GsMCU5.3* displayed decreased expression at 1 h, followed by an increase. These results suggest that *GsMCU* genes may participate in soybean response to carbonate alkaline stress.

To further understand the changes in expression of *GsMCUs* under cold and carbonate alkaline stresses, we predicted stress-related *cis*-acting elements in their promoters (2 kb upstream of the transcription start site) based on the New PLACE database (Table 2). We found that two cold-related *cis*-acting elements, LTR (CCGAAA) and DRE/CRT (TACCGACAT), were observed in the promoters of *GsMCU1.1*, *GsMCU5.1*, *GsMCU6.2*, and *GsMCU6.4*. Moreover, phytohormones play well-documented and critical roles in stress responses (Fahad et al., 2015; Manasa et al., 2021). Several *cis*-elements related to phytohormones have also been found in *GsMCU* promoters, such as ABA (ABRE, core sequence ACGTG) (Gusta et al., 2005), JA (CGTCA, core sequence CGTCA) (Kosova et al., 2012), GA (GARE, core sequence TCTGTTG) (Manasa et al., 2021), and SA (TCA, core sequence CCATCTTTTT) (Miura and Ohta, 2010). Notably, *cis*-elements related to ABA and JA, which have been suggested to play key roles in cold stress, were enriched in the *GsMCU* promoters, indicating that *GsMCU* expression may respond to cold stress via ABA and JA signaling pathways.

**TABLE 2:** Distribution of *cis*-acting elements in the promoters of *GsMCUs*.

Name	Core sequence	Involved in	MCU 1.1	MCU 1.2	MCU 1.3	MCU 1.4	MCU 5.1	MCU 5.2	MCU 5.3	MCU 6.1	MCU 6.2	MCU 6.3	MCU 6.4
LTR	CCGAAA	Cold	1				1				2		
DRE	TACCGACAT	Dehydration, cold, salt											1
ABRE	ACGTG	ABA	3	5	2	1	1			6	4	4	5
CGTCA	CGTCA	JA	2	1	2	1		2	1		2		2
GARE	TCTGTTG	GA			1		2			1		1	1
P-box	CCTTTTG	GA				1			1				
TCA	CCATCTTTTT	SA	1	1	1	2	2			1	1		1

### The Interaction Network of Soybean Mitochondrial Calcium Uniporter Proteins

**FIGURE 9:**
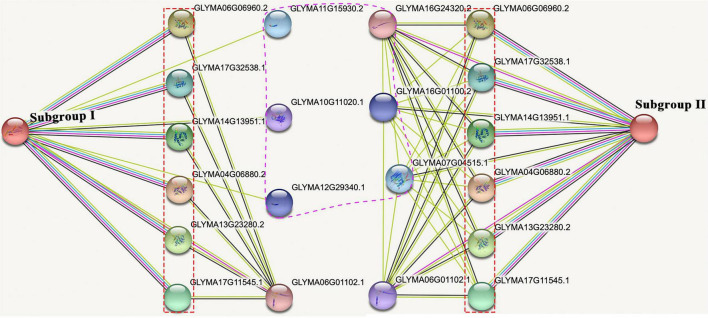
Protein-protein interaction network of soybean MCU proteins. The experimentally determined interacting proteins of the GsMCUs are marked with red boxes. Differences in interacting proteins between the two subgroups are marked by a purple dotted line.

To explore the potential function of *GsMCUs*, we used the STRING database to generate a protein interaction network for each GsMCU. Remarkably, the predicted interacting proteins of GsMCUs within each subgroup were identical. Therefore, we proposed two networks for the GsMCU family (Figure 9): network I (for subgroup I) and network II (for subgroup II). Each network contained 10 interacting partners, among which seven proteins were the same between networks I and II. The same interacting proteins between network I and network II further supported that the GsMCU family was a highly conserved gene family and possibly shared similar molecular and biological functions.

Notably, among the seven proteins shared by networks I and II are six mitochondrial calcium uptake (MICU) proteins that help transport Ca^2+^ along electrochemical gradients by directly interacting with MCUs. Within network I, *Glyma11g15930* and *Glyma12g29340* encode transport protein Sec61 subunit beta, which forms the Sec61 complex for transporting newly synthesized polypeptides into the ER lumen and allows calcium leakage from the ER into the cytosol (Schmutz et al., 2010). *Glyma10g11020* in network I encodes a calcium-dependent protein kinase that senses Ca^2+^ oscillations and mediates Ca^2+^ signal transduction. Within network II, *Glyma16g01100* and *Glyma07g04515* belong to the Ca^2+^ cation antiporter (CaCA) superfamily, which play a vital role in Ca^2+^ ion homeostasis (Table 3). In summary, the investigation of GsMCU-interacting proteins strongly suggests that GsMCUs play a regulatory role in intracellular Ca^2+^ signal transduction.

**TABLE 3:** Annotation of MCU interacting protein in soybean.

	Interacting partner	Functional annotation	Arabidopsis homologous	Function	References
Shared by network I/II	Glyma06g06960	Calcium uptake protein	AT4G32060	Calcium uptake protein 1	Wang and Teng, 2018
	Glyma17g32538	Calcium uptake protein		(MICU)	
	Glyma14g13951	Calcium uptake protein			
	Glyma04g06880	Calcium uptake protein			
	Glyma13g23280	Calcium uptake protein			
	Glyma17g11545	Calcium uptake protein			
	Glyma06g01102	DUF1640, Uncharacterized protein	AT2G16460	DUF1640, Uncharacterized protein	Cheng et al., 2017
Network I-specific	Glyma10g11020	Calcium-dependent protein kinase	AT2G38910	Calcium-dependent protein kinase 20 (CPK20)	Shi et al., 2018
	Glyma11g15930	Transport protein Sec61 subunit beta	AT5G60460	transport protein Sec61 subunit beta	
	Glyma12g29340	Transport protein Sec61 subunit beta			Consortium et al., 2011
Network II-specific	Glyma16g24320	DUF1640, Uncharacterized protein	AT2G16460	DUF1640, Uncharacterized protein	Cheng et al., 2017
	Glyma16g01100	Ca^2+^: cation antiporter (CaCA) family	AT1G54115	Cation calcium exchanger 4 (CCX4)	Fujikura et al., 2020
	Glyma07g04515	Ca^2+^: cation antiporter (CaCA) family			

## Discussion

It has been suggested that the MCU genes regulate Ca^2+^ signal transduction and participate in diverse physiological processes. Although mammalian MCU genes have been well characterized, the biological functions and regulatory roles of plant MCUs are unknown. Here, we systematically investigated the wild soybean MCU family genes and provided clues regarding their potential function in soybean development and abiotic stress responses.

### Soybean Mitochondrial Calcium Uniporter Proteins Are Highly Conserved

In this study, we identified a total of 11 *GsMCU* genes and suggested that the GsMCU family was a highly conserved family. First, all *GsMCUs* contained one long intron at a similar position, indicating a conserved gene structure (Figure 3). Secondly, all GsMCU proteins harbored one conserved MCU domain, with two predicted transmembrane regions (Figure 4A). It has been suggested that the pentamerization of transmembrane regions is required for the formation of a functional channel (Oxenoid et al., 2016). MEME analysis further showed high similarity in conserved motif organization of the MCU domain, as well as the N- and C-terminal regions (Figure 4B), suggesting a conserved protein structure. Previous studies reported that the N-terminal domain was important for the Ca^2+^ uptake activity of the MCU proteins in human (Lee et al., 2015). Further studies are need to identify the function of conserved domains in Ca^2+^ transport activity. Third, based on the PGDD database, we identified a total of 21 duplicated pairs among 11 *GsMCU* genes, possibly due to whole-genome duplication of soybean during evolution (Figure 2). Synteny analysis also identified several duplicated MCU gene pairs in soybean, alfalfa, and Arabidopsis (Supplementary Figure 1). Similarly, whole-genome duplication is the main driving force for pear MCU family expansion (Wang et al., 2018). Moreover, MCUs from different plant species were clustered into two subgroups and showed similar phylogenetic relationships (Figure 1). In the phylogenetic tree, the GsMCUs appeared in pairs. Previous studies have also reported that MCUs from Arabidopsis and pear were clustered into two subgroups (Stael et al., 2012; Wang et al., 2018). Finally, GsMCUs within each subgroup shared the same interacting proteins in the STRING network prediction (Figure 9). In human, MICU1 is suggested to be associated with the mitochondrial inner membrane and is required for high-capacity mitochondrial Ca^2+^ uptake (Perocchi et al., 2010). In Arabidopsis, MICU was demonstrated to regulate mitochondrial Ca^2+^ dynamics in intact plants (Wagner et al., 2015). Therefore, it is important to validate the consensus protein interaction of GsMCU family members. Taken together, our results strongly demonstrate that the GsMCU family is highly evolutionarily conserved.

### Mitochondrial Calcium Uniporter Genes Are Involved in Regulating Soybean Development

Although the MCU family is conserved, they display a certain degree of diversity in subcellular localization and tissue-specific expression characteristics. In Arabidopsis, AtMCU1–AtMCU5 localize in the mitochondria (Teardo et al., 2017; Nomura and Shiina, 2021) and AtMCU6 localizes in the chloroplast envelope (Carraretto et al., 2016; Teardo et al., 2019). Therefore, further studies are required to verify the mitochondria localization of GsMCU1s and GsMCU5s, as well as the chloroplast localization of GsMCU6s. Moreover, our studies also showed that *GsMCU6.2* and *GsMCU6.3* from the MCU6 branch were highly expressed in stems and flowers, respectively, while members from the MCU5 branch were highly expressed in seeds and leaves (Figure 6). Similarly, *AtMCU* genes also show specific expression characteristics in different tissues (Teardo et al., 2017, 2019). For example, *AtMCU1* displayed the highest expression in roots and an intermediate expression in young leaves, fully expanded leaves, old leaves, and flowers, but no expression (below the detection limit) in seeds (Teardo et al., 2017). *AtMCU4* also showed the highest expression in roots (Teardo et al., 2017). *AtMCU6* is expressed in all tissues, with higher levels in the mature leaves (Teardo et al., 2019). Furthermore, *PbrMCUs* also exhibited different expression profiles in different parts of “Housui” pear fruit (Wang et al., 2018). The diversity of MCUs in terms of subcellular localization and tissue expression might be the main driving force for their potential function specialization in regulating plant growth and development.

Several studies have reported the role of MCU genes in plant development. Interestingly, both *AtMCU1* knockout and overexpression caused shorter primary roots under restrictive growth conditions (Teardo et al., 2017). *AtMCU2* knockout impairs Arabidopsis pollen tube germination and growth (Selles et al., 2018). Arabidopsis plants lacking *AtMCU6* exhibit constitutive stomatal closure (Teardo et al., 2019). Furthermore, three MCU genes are involved in pear ripening (Wang et al., 2018). In future, genetic data regarding *GsMCUs* overexpression or knockout will be of great importance for understanding their biological roles during soybean growth and development.

### Expression of *Glycine soja* MCUs Reveals Their Potential Roles in Response to Cold and Carbonate Alkaline Stress

Ca^2+^ signal transduction is of great importance in plant responses to various abiotic stresses including drought, salt and cold. In this study, we identified that six *GsMCUs* responded to cold stress (Figure 7), and nine *GsMCUs* showed increased expression under carbonate alkaline treatment (Figure 8). In particular, the MCU6 branch genes were upregulated under both cold and carbonate alkaline stresses, while *GsMCU5s* were downregulated. This difference indicates potential functional specialization of subgroup II MCUs. Members within the MCU6 and MCU5 branches, as well as three subgroup I members, showed roughly the same expression pattern under stress treatment, suggestive of possible functional redundancy. A recent study has reported that *AtMCU6* regulates osmotic stress responses (Teardo et al., 2019). *AtMCU6* knockout plants displayed increased resistance to long-term water deficits and improved recovery after re-watering. Therefore, functional validation of *GsMCUs* by using overexpressing or gene-editing soybean is particularly important for investigating their precise biological roles and molecular basis in regulating the cold and carbonate alkaline stress tolerance.

Furthermore, we observed several stress-related *cis*-acting elements in the promoters of *GsMCUs*, including two cold-related *cis*-acting elements, LTR (CCGAAA) and DRE/CRT (TACCGACAT), as well as ABA (ABRE, core sequence ACGTG) and JA (CGTCA, core sequence CGTCA) responsive *cis*-elements (Table 2). A previous study showed that *PbrMCU* expression is affected by another type of phytohormone, ethylene (Wang et al., 2018). Hence, more experiments are need to verify the hormone induced expression of *GsMCUs*, and the binding of key transcription factors, for example CBF/DREB1s, to *GsMCUs* promoters. Furthermore, the genetic evidence is also required in future to confirm whether *GsMCUs* participate in cold and carbonate alkaline stress via the ABA and JA signaling pathways.

## Conclusion

In this study, we identified the MCU family genes in wild soybean and investigated their expression under cold and carbonate alkaline stresses. Eleven *GsMCUs* were identified and clustered into two subgroups. Gene duplication was detected in the *GsMCU* family, and the duplicated pairs displayed a similar exon-intron architecture. All GsMCU proteins contained one conserved MCU domain and two transmembrane helices, showing high conservation in protein sequence and structure. Moreover, all *GsMCUs* were expressed ubiquitously in different tissues and organs, and six and nine GsMCUs were differentially expressed under cold and carbonate alkaline stress, respectively. Our results provide valuable information about the soybean MCU family, which will facilitate functional analysis of *GsMCUs* in the future.

## Data Availability Statement

Publicly available datasets were analyzed in this study. This data can be found here: The datasets for the RNAseq experiments can be found on NCBI GEO (Accessions # GSE117686 and GSE17883) (https://www.ncbi.nlm.nih.gov/geo/query/acc.cgi?acc=GSE117686 and https://www.ncbi.nlm.nih.gov/geo/query/acc.cgi?acc=GSE17883).

## Author Contributions

XS and KY guided the design of the experiment and revised the manuscript. JL and MS conducted data analysis and manuscript writing. YL finished plant material handling. All authors reviewed and agreed to the published version of the manuscript.

## Conflict of Interest

The authors declare that the research was conducted in the absence of any commercial or financial relationships that could be construed as a potential conflict of interest.

## Publisher’s Note

All claims expressed in this article are solely those of the authors and do not necessarily represent those of their affiliated organizations, or those of the publisher, the editors and the reviewers. Any product that may be evaluated in this article, or claim that may be made by its manufacturer, is not guaranteed or endorsed by the publisher.

## Footnotes

1
https://phytozome-next.jgi.doe.gov

2
http://hmmer.janelia.org/

3
https://www.ncbi.nlm.nih.gov/Structure/cdd/wrpsb.cgi

4
http://pfam.sanger.ac.uk/

5
http://www.expasy.org/

6
http://www.cbs.dtu.dk/services/TMHMM/

7
https://orthovenn2.bioinfotoolkits.net/home

8
http://gsds.gao-lab.org

9
https://www.ncbi.nlm.nih.gov/Structure/bwrpsb/bwrpsb.cgi

10
http://meme-suite.org/tools/meme

11
https://cn.string-db.org/cgi/input.pl

12
https://www.dna.affrc.go.jp/PLACE/?action=newplace
