# Supplementary material for: Genome-Wide Identification of Wild Soybean Mitochondrial Calcium Uniporter Family Genes and Their Responses to Cold and Carbonate Alkaline Stresses
Source: Front Plant Sci. 2022 May 3;13:867503. doi: 10.3389/fpls.2022.867503 (PMC9111538; doi:10.3389/fpls.2022.867503)
Supplement: Supplementary file 1 [file Data_Sheet_1.docx]

Supplementary Material

## Supplementary Figures

###
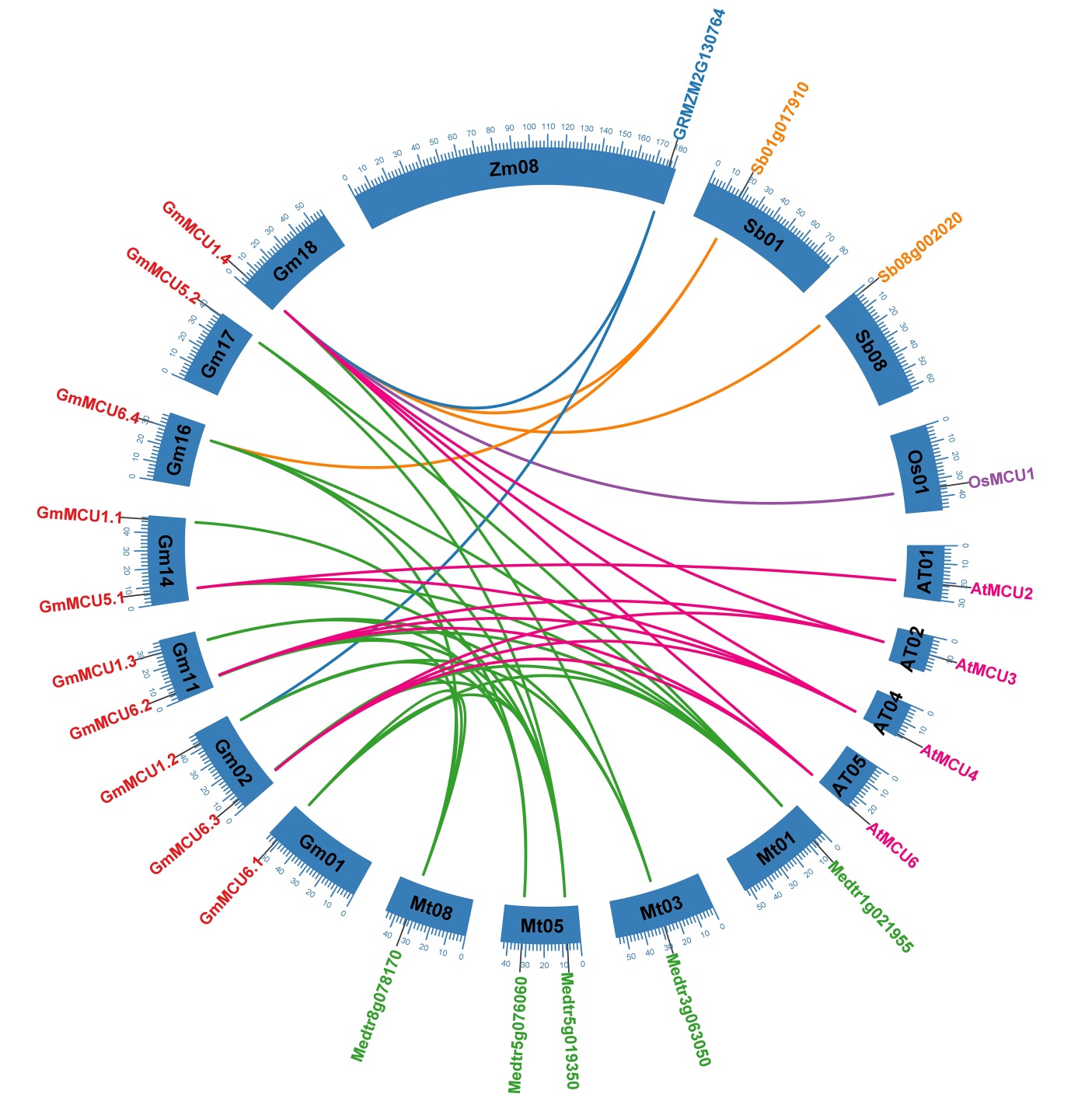


### Supplemental Figure S1. Distribution and duplication of the MCU genes in soybean, Arabidopsis, rice, alfalfa, sorghum and maize.

The red, pink, purple, green, yellow, and blue words within the chromosomes represent the locations of GmMCUs, AtMCUs, OsMCUs, MtMCUs, SbMCUs, and ZmMCUs, respectively. The pink, purple, green, yellow, and blue lines indicate the duplication relationships between GmMCUs and AtMCUs, OsMCUs, MtMCUs, SbMCUs, and ZmMCUs, respectively.

### Supplemental Table S1. Numbers and subgroups of MCU genes in Arabidopsis, rice and soybean.

| **Species** | **Subgroup I** | **Subgroup II** | **In total** |
| --- | --- | --- | --- |
| *Arabidopsis thaliana* | 2 | 4 | 6 |
| *Oryza sativa* | 3 | 2 | 5 |
| *Glycine max* | 4 | 7 | 11 |
| *Glycine soja* | 4 | 7 | 11 |

### Supplemental Table S2. Information about gene duplication in cultivated soybean.

| **Gene duplication** | **Chromosome** | **Similarity** | **Duplicate type** | **Ka** | **Ks** | **Ka/Ks** | **Duplicated time / Mya** |
| --- | --- | --- | --- | --- | --- | --- | --- |
| GmMCU1.1/GmMCU1.4 | 14/18 | 82.2% | segmental duplication | 0.186 | 0.7303 | 0.2547 | 59.8607 |
| GmMCU1.2/GmMCU1.3 | 2/11 | 79.4% | segmental duplication | 0.1406 | 0.6809 | 0.2065 | 55.8115 |
| GmMCU1.2/GmMCU1.1 | 2/14 | 91.9% | segmental duplication | 0.0627 | 0.1459 | 0.4297 | 11.9590 |
| GmMCU1.2/GmMCU1.4 | 2/18 | 80.6% | segmental duplication | 0.189 | 0.8181 | 0.2310 | 67.0574 |
| GmMCU1.3/GmMCU1.1 | 11/14 | 84.0% | segmental duplication | 0.1399 | 0.6243 | 0.2241 | 51.1721 |
| GmMCU1.3/GmMCU1.4 | 11/18 | 98.2% | segmental duplication | 0.0135 | 0.1334 | 0.1012 | 10.9344 |
| GmMCU5.1/GmMCU6.4 | 14/16 | 65.1% | segmental duplication | 0.5397 | 3.9324 | 0.1372 | 322.3279 |
| GmMCU5.1/GmMCU5.2 | 14/17 | 92.0% | segmental duplication | 0.0735 | 0.2763 | 0.2660 | 22.6475 |
| GmMCU6.1/GmMCU6.3 | 1/2 | 79.3% | segmental duplication | 0.2386 | 0.9 | 0.2651 | 73.7705 |
| GmMCU6.1/GmMCU6.2 | 1/11 | 95.4% | segmental duplication | 0.045 | 0.1284 | 0.3505 | 10.5246 |
| GmMCU6.1/GmMCU5.1 | 1/14 | 73.5% | segmental duplication | 0.5146 | 2.5688 | 0.2003 | 210.5574 |
| GmMCU6.1/GmMCU6.4 | 1/16 | 79.2% | segmental duplication | 0.2583 | 0.973 | 0.2655 | 79.7541 |
| GmMCU6.1/GmMCU5.2 | 1/17 | 70.0% | segmental duplication | 0.5056 | 1.8268 | 0.2768 | 149.7377 |
| GmMCU6.2/GmMCU5.1 | 11/14 | 72.2% | segmental duplication | 0.5101 | 2.6501 | 0.1925 | 217.2213 |
| GmMCU6.2/GmMCU6.4 | 11/16 | 78.5% | segmental duplication | 0.2782 | 1.0647 | 0.2613 | 87.2705 |
| GmMCU6.2/GmMCU5.2 | 11/17 | 70.0% | segmental duplication | 0.5387 | 2.0621 | 0.2612 | 169.0246 |
| GmMCU6.3/GmMCU6.2 | 2/11 | 79.5% | segmental duplication | 0.2658 | 0.9395 | 0.2829 | 77.0082 |
| GmMCU6.3/GmMCU5.1 | 2/14 | 66.0% | segmental duplication | 0.4839 | 0 |  |  |
| GmMCU6.3/GmMCU6.4 | 2/16 | 97.0% | segmental duplication | 0.0332 | 0.2101 | 0.1580 | 17.2213 |
| GmMCU6.3/GmMCU5.2 | 2/17 | 73.1% | segmental duplication | 0.4765 | 2.6803 | 0.1778 | 219.6967 |
| GmMCU6.4/GmMCU5.2 | 16/17 | 73.6% | segmental duplication | 0.551 | 2.7837 | 0.1979 | 228.1721 |

### Supplementary Table S3. Gene specific primers for quantitative real-time PCR analyses.

| **Gene name** | **Primer sequence (5’-3’)** | **Sequence Length (bp)** |
| --- | --- | --- |
| *GsMCU1.1* | Forward: GTTCATCCTTTTGATCCTTTCAGGG | 116 |
|  | Reverse: GCAGGGGATGAAAGTAAAGTTGAGG |  |
| *GsMCU1.2* | Forward: GAGATTGGTGAATGTGGAATCG | 190 |
|  | Reverse: CAGATAGACCTTGTCTCGGAAG |  |
| *GsMCU1.3* | Forward: AGAGCTGTTCCACTGGTGCT | 250 |
|  | Reverse: CGTAGCCTATGACCAACCCAG |  |
| *GsMCU1.4* | Forward: GCAAGACACCTTTACATGCTAG | 158 |
|  | Reverse: CACTAAAACAAGGGGAACAAGG |  |
| *GsMCU5.1* | Forward: GGAAACTGTTATCGAAGCGTTT | 267 |
|  | Reverse: CGATGTCGTTGATGTCTTTGAG |  |
| *GsMCU5.2* | Forward: ATGGTGCTTCAGAAACTGTTATC | 216 |
|  | Reverse: GAAATCCGGGAAACGTGCCGGAC |  |
| *GsMCU5.3* | Forward: CCAACTATGCAACAACAAGTCA | 89 |
|  | Reverse: AAAAACCGCAAGAGAAATCCTC |  |
| *GsMCU6.1* | Forward: GTTCATTGGGATCTGCGATGAG | 191 |
|  | Reverse: CTCTTCTTACCGACTCGTGCG |  |
| *GsMCU6.2* | Forward: CACCAAAGTGTCATCACAATCCCTC | 269 |
|  | Reverse: CCACCTTTAGCAATTTTCTGGCATC |  |
| *GsMCU6.3* | Forward: CCGCAGAGTTGCATAAGCTATTCAG | 181 |
|  | Reverse: CAGGGAGTAGAGATTGGATGGTTTG |  |
| *GsMCU6.4* | Forward: CAAGAAAATGAACGCCAAATCG | 171 |
|  | Reverse: CCTGTGTACAACTTAGTGCAAC |  |
| *F_box* | Forward: CTAATGGCAATTGCAGCTCTC | 93 |
|  | Reverse: AGATAGGGAAATTGTGCAGGT |  |
